# ERBB3 binding protein 1 promotes the progression of malignant melanoma through activation of the Wnt/ β-catenin signaling pathway

**DOI:** 10.1186/s12935-022-02473-6

**Published:** 2022-01-29

**Authors:** Yanqiu Bao, Jingshu Cui, Yuyang Yue, Shuxia Cao, Xiangdan Li, Lan Liu

**Affiliations:** 1 Department of Dermatology, Yanbian University Hospital, Jilin, 133000 China; 2 Department of Pathology, Yanbian University Hospital, Jilin, 133000 China; 3 Center of Morphological Experiment, Medical College of Yanbian University, Jilin, 133000 China

**Keywords:** Ebp1, Malignant melanoma, EMT, Wnt/β-catenin

## Abstract

**Background:**

Malignant melanoma (MM) is highly metastatic and has the highest mortality rate in patients with skin cancer. The ERBB3 binding protein 1 (Ebp1) has been linked to the onset and progression of a number of malignancies. However, the role of Ebp1 in MM has not yet been reported.

**Methods:**

Multiple databases were analyzed for comparing the expression of Ebp1 in normal skin and MM. Ebp1 expression was knocked down in A375 and B16 cells, and the impact of Ebp1 on the cell growth was tested by CCK-8, plate clone colony, and cell cycle assays. Scratch, transwell, and in vivo caudal vein lung metastasis tests were also used to confirm the effects of Ebp1 on melanoma cells migration, invasion, and metastasis. Furthermore, the possible molecular mechanism of Ebp1 was predicted by set enrichment analysis and verified by western blotting.

**Results:**

Ebp1 expression was substantially higher in MM than it was in normal skin, and Ebp1 was linked to the clinical stage and lymph node metastases of patients with MM. Knockdown of Ebp1 inhibited cell proliferation, migration, and invasion. In vivo experiments further verified that the knockdown of Ebp1 had an obvious inhibitory effect on lung metastasis in nude mice. Knockdown of Ebp1 reduced vimentin, N-cadherin, slug, and snail expression while increasing E-cadherin expression. Furthermore, knockdown of Ebp1 reduced the expression of β-catenin, as well as its downstream targets CyclinD1 and p-GSK3β; however, a Wnt/β-catenin agonist could reverse this effect.

**Conclusion:**

Ebp1 may promote the proliferation and metastasis of melanoma cells through activation of the Wnt/β-catenin pathway.

**Graphical Abstract:**

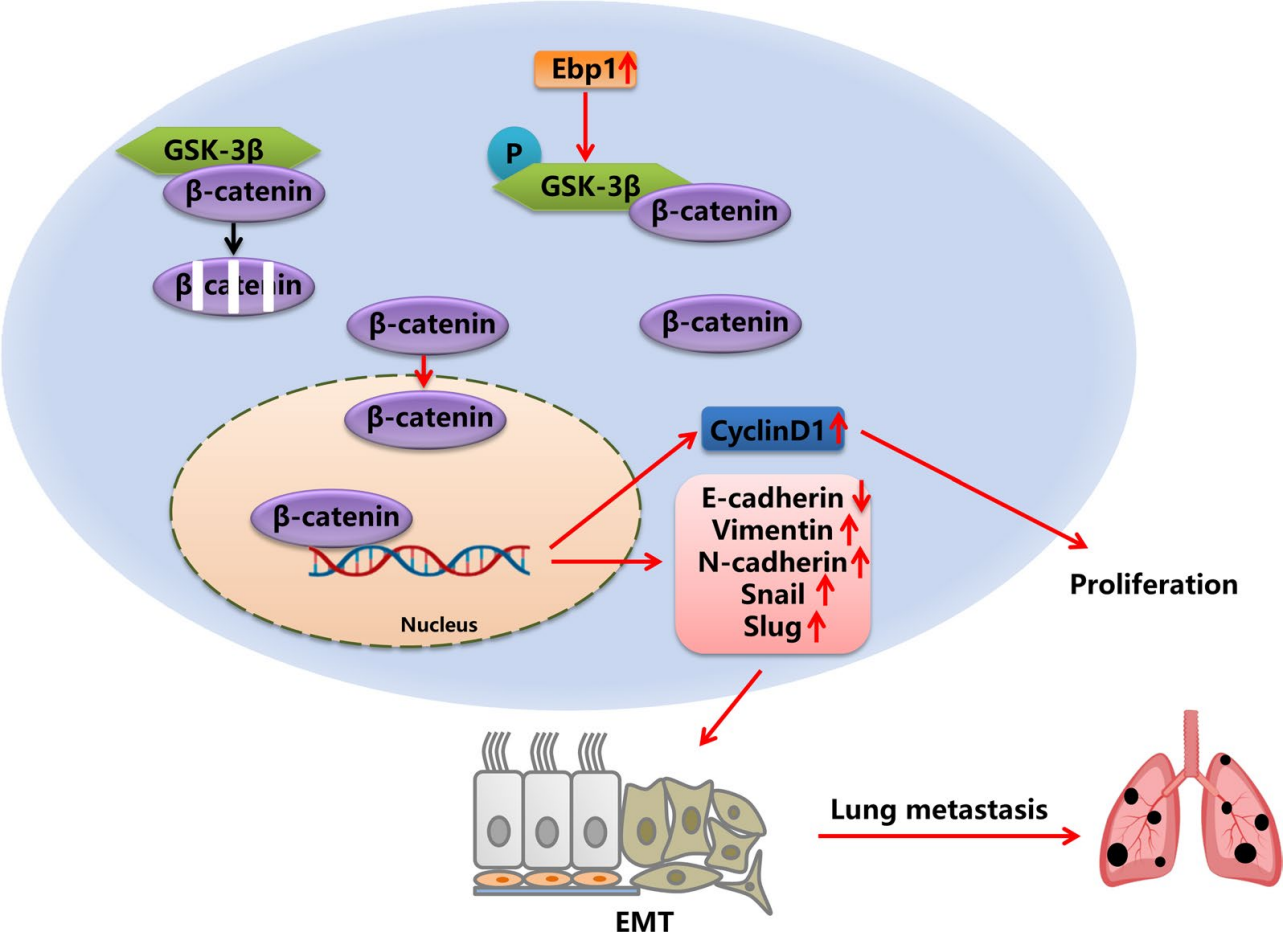

## Introduction

As one of the deadliest cancers, malignant melanoma (MM) is a highly malignant tumor and difficult to treat when diagnosed in the late stage. Approximately 75% of skin cancer patients die of melanoma, although it accounts for approximately 2% of all skin cancers; thus, MM is the main cause of death from skin cancer [1]. Meanwhile, the incidence rate of melanoma has been increasing sharply with the affected population gradually becoming younger [2]. The principal treatment for early stage melanoma is surgery; however, 40–60% of high-risk cases experience recurrence and metastasis [3], and patients with metastatic melanoma have a five-year survival rate of only 10% [4]. In the past decades, targeted therapy has emerged as the main treatment for various cancers, and new targets have been discovered one after the other. As a result, it's critical to look at potential molecular therapy targets for MM.

Epithelial mesenchymal transition (EMT) is a key process in tumor cell metastasis. EMT has been shown to play a significant role in progression and metastasis of melanomas [5]. EMT is regulated by a variety of signaling pathways, among which is the Wnt/β-catenin signaling pathway, one of the most classic pathways [6, 7]. The Wnt/β-catenin pathway is closely related to the occurrence, progression, metastasis, and drug resistance of cancer cells. It plays an important role in the development of many cancers, such as colorectal cancer, melanoma, breast cancer, lung cancer, and leukemia [8].

Proliferation associated protein 2G4 (PA2G4), also known as the ERBB3 binding protein 1 (Ebp1), is a widely expressed multifunctional protein [9]. There are two types of p48 and p42. Both subtypes play regulatory roles in cell growth and differentiation. However, these two subtypes play opposite roles in different tissues [10]. In a number of cancerous tumors, the expression of the long subtype Ebp1 p48 is significantly increased, such as glioblastoma multiforme (GBM) [11], colon cancer [12], hepatocellular carcinoma [13], and acute myeloid leukemia [14]. The expression level of Ebp1 p48 is also closely linked to cancer patients’ clinical prognosis. Studies have shown that PA2G4 is an independent prognostic factor in breast cancer by means of 2-nitrobenzenesulfenyl chloride/two-dimensional gel electrophoresis/mass spectrometry (NBS/2DE /MS) protein quantification [15]. In addition, GBM patients with increased Ebp1 p48 expression had a significantly worse prognosis than those with lower expression [11]. Since the longer subtype of Ebp1 p48 is the main expressed form, its carcinogenic function deserves more attention. Nevertheless, the tumor suppressive effect of the shorter subtype Ebp1 p42 in other cancers cannot be ignored [16–18].

In this study, we explored the mechanism by which Ebp1 promotes the proliferation and metastasis of melanoma cells, as well as the expression of Ebp1 in melanoma tissues. Moreover, we also looked into the effect of Ebp1 on the Wnt/β-catenin pathway.

## Materials and methods

### Cell culture

Human keratinocytes HaCaT, human melanoma cells A375 and murine melanoma cells B16 were purchased from the National Collection of Authenticated Cell Cultures. The above cells were cultured in DMEM medium (Gibco) with 10% FBS (Bioind) and 1% penicillin–streptomycin solution (Bioind) at 37 ℃ incubator supplemented with 5% CO_2_. Cell lines were tested monthly for mycoplasma and validated via STR testing.

### Cell transfection

A375 and B16 cells were transduced with the shRNA lentivirus targeting Ebp1 (TRCN050413, target sequence: CCGGCCACCAGCATTTCGGTAAATACTCGAGTATTTACCGAAATGCTGGTGGTTTTTG, Sigma-Aldrich) or the non-silencing control (TRCN0286798 target sequence: CCGGCCTGGTCGTGACCAAGTATAACTCGAGTTA TACTTGGTCACGACCAGGTTTTTG, Sigma-Aldrich) and then selected with 1 μg/ml puromycin for 7 days. The transfection efficacy was determined by western blot.

### Cell growth assay

For cell proliferation analysis, A375 and B16 cells transduced to express sh-NC and sh-Ebp1 were seeded on 96 well plates at 1 × 10^3^ / well and cultured for 0, 1, 2, 3, 4, 5 and 6 days, cells were cultured for 1 h at 37 ℃ with 10ul of CCK-8 solution. Absorbance value was measured at 450 nm.

### Colony formation assay

Gene transduced A375 and B16 cells were seeded into 6-well plates (2 × 10^3^ cells/ well) and cultured for 14 days. Then the cells were first washed twice with PBS solution and fixed with 4% paraformaldehyde (Solarbio) for 15 min. The cells were stained using Giemsa stain (Solarbio).

### Flow cytometry

A375 and B16 cells were fixed in 70% ethanol for 18 h at 4 °C, washed with PBS, and incubated with Propidium Iodide for 30 min. Cells were analyzed by flow cytometer (BD Biosciences).

### Wound healing assay

Gene transduced A375 and B16 cells were seeded on 6 well plates, grown to near confluence, and incubated with serum-free media overnight. Then cells were wounded with a 10-μl pipette tip. Images were taken at 0 and 48 h after wounding.

### Transwell assay

The A375 and B16 cells were trypsinized and resuspended in DMEM without serum, and 300 μl cell suspension with 2 × 10^5^ cells administered to the top chamber, 600ul complete DMEM was added to the deeper well. 24 h later, the cell was fixed for 15 min and stained with Giemsa, then washed with ddH2O and photographed.

### Western blot

Cells were grown to approximately 90% confluence in the 10%FBS/DMEM medium Protein lysates were prepared by RIPA buffer. For Western blotting, proteins (20 μg/sample) were separated by different polyacrylamide gel electrophoresis, transferred onto PVDF membrane, and incubated with the primary antibodies overnight. Then the blots were incubated with HRP-conjugated secondary antibodies (Boster Biological Technology), detected with the X-ray film and imaged. The antibodies used in the experiment were shown in Table 1.

**Table 1:** Antibodies used in the experiment

Primary antibody	Concentration	Company	Second antibody
Ebp1 (N-terminus) (WB)	1:1000	Millipore	Goat anti-rabbit IgG
Vimentin (C-20) (WB)	1:1000	Santa Cruz	Rabbit anti-goat IgG
N-cadherin (13A9) (WB)	1:1000	Santa Cruz	Goat anti-mouse IgG
Snail (ab53519) (WB)	1:1000	Abcam	Goat anti-rabbit IgG
E-cadherin (S-17) (WB)	1:1000	Santa Cruz	Rabbit anti-goat IgG
Slug (A-7) (WB)	1:1000	Santa Cruz	Goat anti-mouse IgG
β-catenin (E-5) (WB)	1:1000	Santa Cruz	Goat anti-mouse IgG
p-GSK-3β (Ser 9) (WB)	1:1000	Santa Cruz	Rabbit anti-goat IgG
cyclinD1 (A-12) (WB)	1:1000	Santa Cruz	Goat anti-mouse IgG
β-actin (C4) (WB)	1:1000	Santa Cruz	Goat anti-mouse IgG
Vimentin (C-20) (IF)	1:200	Santa Cruz	TRITC-rabbit anti-goat IgG
E-cadherin (S-17) (IF)	1:200	Santa Cruz	TRITC-rabbit anti-goat IgG

### Immunofluorescence

Cells were seeded on coverslips and cultured until 70% confluence, then permeabilized in PBS containing 0.4% Triton, fixed in PBS with 4% paraformaldehyde and blocked with 1% BSA for 1 h. The primary antibody was added and incubated overnight, then incubated with secondary antibody for 1 h and stained with DAPI. The cells were visualized and imaged with a laser scanning microscope (Nikon, Japan).

### Animal studies

Animal studies were performed in compliance with Yanbian University Ethics Committee. 4–6 weeks old female nude mice were purchased from the Experimental animal center of Yanbian University. Animals (n = 6/group) were injected with 100 µl of solution containing 5 × 10^6^ B16 cells (sh-NC; sh-Ebp1) via the caudal vein. 3 weeks later, the mice were sacrificed, and the lung tissues were collected.

### H&E staining

Lung tissues from mice were fixed in 10% formalin, dehydrated in graded ethanol, then embedded in paraffin, cut into 4um thickness slices. Slices were baked in an oven at 56 ℃ overnight and stained with H&E Stain Kit (Solarbio, Beijing, China).

### Statistical analysis

The two-tailed multiple Student’s t-tests were used to determine significant differences and Kaplan–Meier was used to estimate the survival function. Computations were performed using SPSS 26.0 (IBM, USA) or GraphPad Prism 8. The data are expressed as the means ± SD and all p values less than 0.05 were considered statistically significant. All the experiments were repeated at least three times.

## Result

### Ebp1 is highly expressed in melanoma, which is linked to the progression of melanoma

**Fig. 1:**
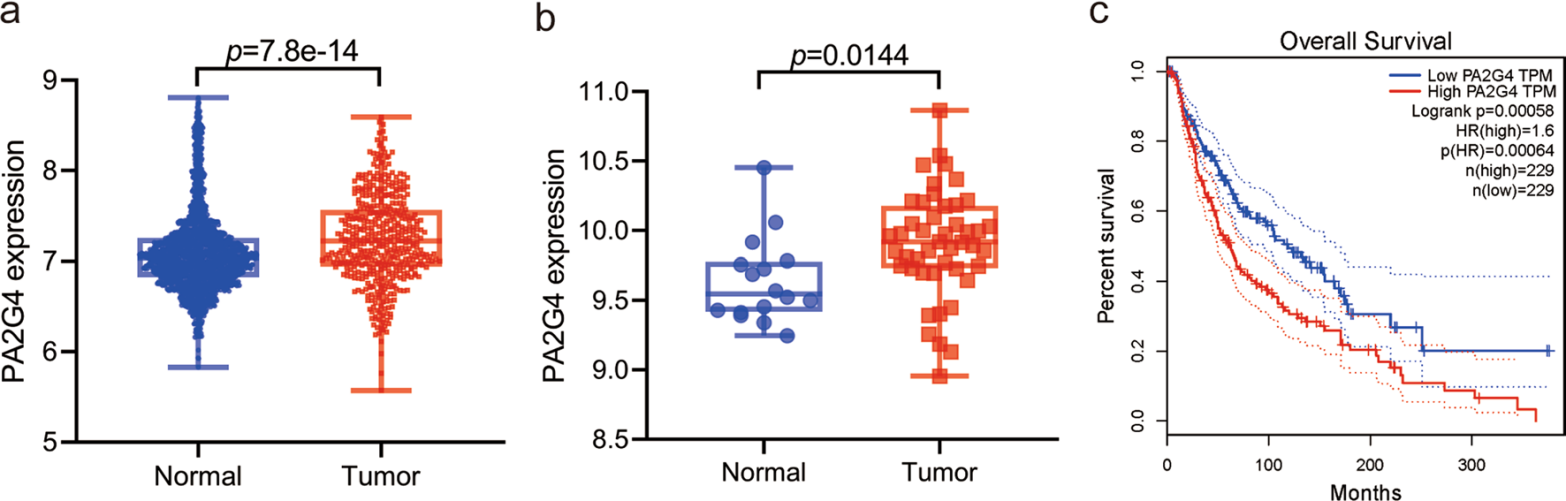
Ebp1 is highly expressed in malignant melanoma and is closely related to prognosis. **a** 1809 normal skin tissue samples were analyzed in the GTEx database, the expression of Ebp1 was increased in 470 malignant melanoma tissue samples in the TCGA database. **b** The expression of Ebp1 was analyzed in tumor tissues of melanoma compared with normal skin tissue from the GEO database. **c** Kaplan–Meier analysis revealed high expression of Ebp1 correlates to poor overall survival in patients with malignant melanoma

We used the Cancer Genome Atlas (TCGA) database to obtain the expression data of 470 patients with MM and downloaded the expression data of 1809 normal skin samples from the Genotype Tissue Expression (GTEx) database. Ebp1 expression was substantially higher in MM tissues than in normal skin tissues (Fig. 1a). To further confirm this result, we selected the GSE5605 dataset from the Gene Expression Omnibus (GEO) database and discovered that Ebp1 expression was considerably higher in MM tissues than in normal skin tissues (Fig. 1b). In addition, the expression of Ebp1 was linked to the clinical stage of MM and lymph node metastases (Table 2). We further analyzed the effect of Ebp1 on the prognosis of patients with MM by Kaplan Meier analysis and discovered that individuals with high Ebp1 expression had a considerably lower overall survival time than patients with low Ebp1 expression (Fig. 1c).

**Table 2:** Summary of correlation of Ebp1 expression with clinicopathological of MM

Clinical characteristics	N	Ebp1		*P* value
		Low(< 0.5)	High(≥ 0.5)	
*Age(years)*				
≤ 60	251	132	119	0.205
> 60	210	98	112	
*Gender*				
Female	179	97	82	0.154
Male	291	138	153	
*Clinical stage*				
Stage I + II	216	97	119	0.026
Stage III + IV	195	109	86	
*T stage*				
T1 + T2	119	62	57	0.415
T3 + T4	244	116	128	
*N stage*				
N0	234	102	132	0.018
N1 + 2 + 3	179	99	80	
*M stage*				
M0	417	209	208	0.326
M1	25	10	15	

### Knockdown of Ebp1 inhibited the proliferation of melanoma cells

**Fig. 2:**
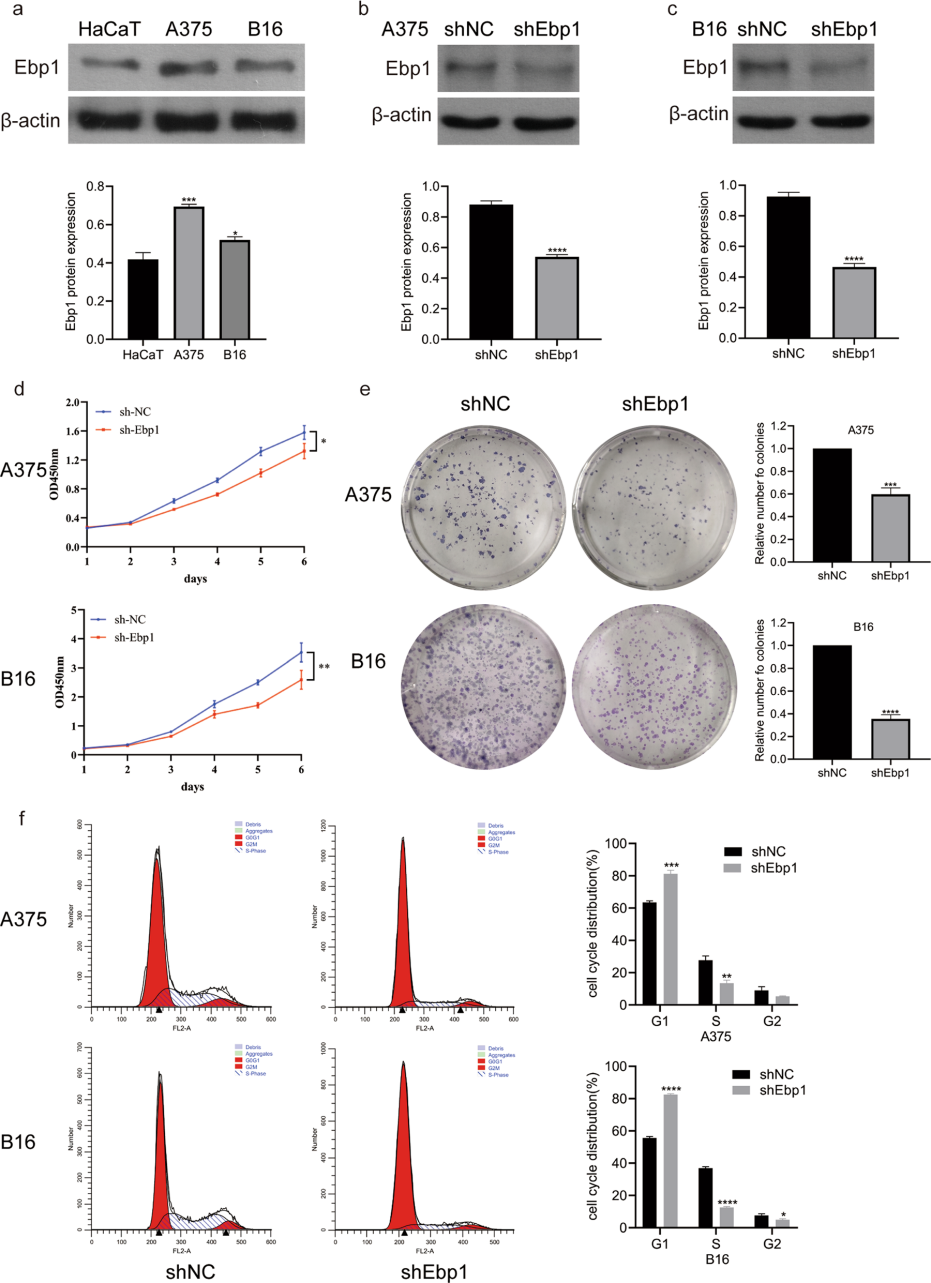
Ebp1 knockdown suppresses the proliferation of malignant melanoma. **a** Ebp1 p48 was expressed in the HacaT, A375 and B16 cells (^***^p < 0.001, ^*^p < 0.05, vs. HacaT). **b**, **c** Ebp1 was knocked down in A375 and B16 cells and detected by Western blotting (^****^p < 0.0001, vs. shNC). **d** Cell proliferation was measured by CCK-8 assay (^**^p < 0.01, ^*^p < 0.05, vs. shNC). **e** Ebp1 knockdown suppressed the colony-forming capacity of A375 and B16 cells by colony-formation assay (^***^p < 0.001, ^****^p < 0.0001, vs. shNC). **f** Effects of Ebp1 knockdown on cell cycle profile in A375 and B16 cells using flow cytometry (^****^p < 0.0001, ^***^p < 0.001, ^**^p < 0.01, ^*^p < 0.05, vs. shNC)

To learn more about the biological function of Ebp1 in melanoma cells, we first detected the expression of Ebp1 in HaCaT, A375, and B16 melanoma cell lines. Ebp1 expression in HaCaT cells was lower than in A375 and B16 cells, according to Western blotting (Fig. 2a). Since Ebp1 was substantially expressed in these cells, we stably knocked down Ebp1 in A375 and B16 cells using an RNA interference lentivirus (Fig. 2b, c). On performing the CCK-8 assay, it was found that the growth of melanoma cells with knockdown of Ebp1 was significantly slower than that of melanoma cells without knockdown of Ebp1 (Fig. 2d). The plate colony formation assay further showed that the ability of melanoma cells with knockdown of Ebp1 to form colonies was considerably weaker than that of the non-knockdown group (Fig. 2e). Based on the results of the cell viability and clonogenic assays, we preliminarily determined that the knockdown of Ebp1 had an inhibitory effect on the proliferation of A375 and B16 cells. To determine whether Ebp1 affects the growth cycle of melanoma cells, we performed cell cycle detection. The percentage of melanoma cells in the G1 phase with Ebp1 knockdown was much greater than in the non-knockdown group, indicating that the cells were inhibited in the G1 phase (Fig. 2f). Therefore, knocking down Ebp1 blocked the cells in the G1 phase, reducing melanoma cell growth considerably.

### Knockdown of Ebp1 inhibited the metastasis of melanoma cells

**Fig. 3:**
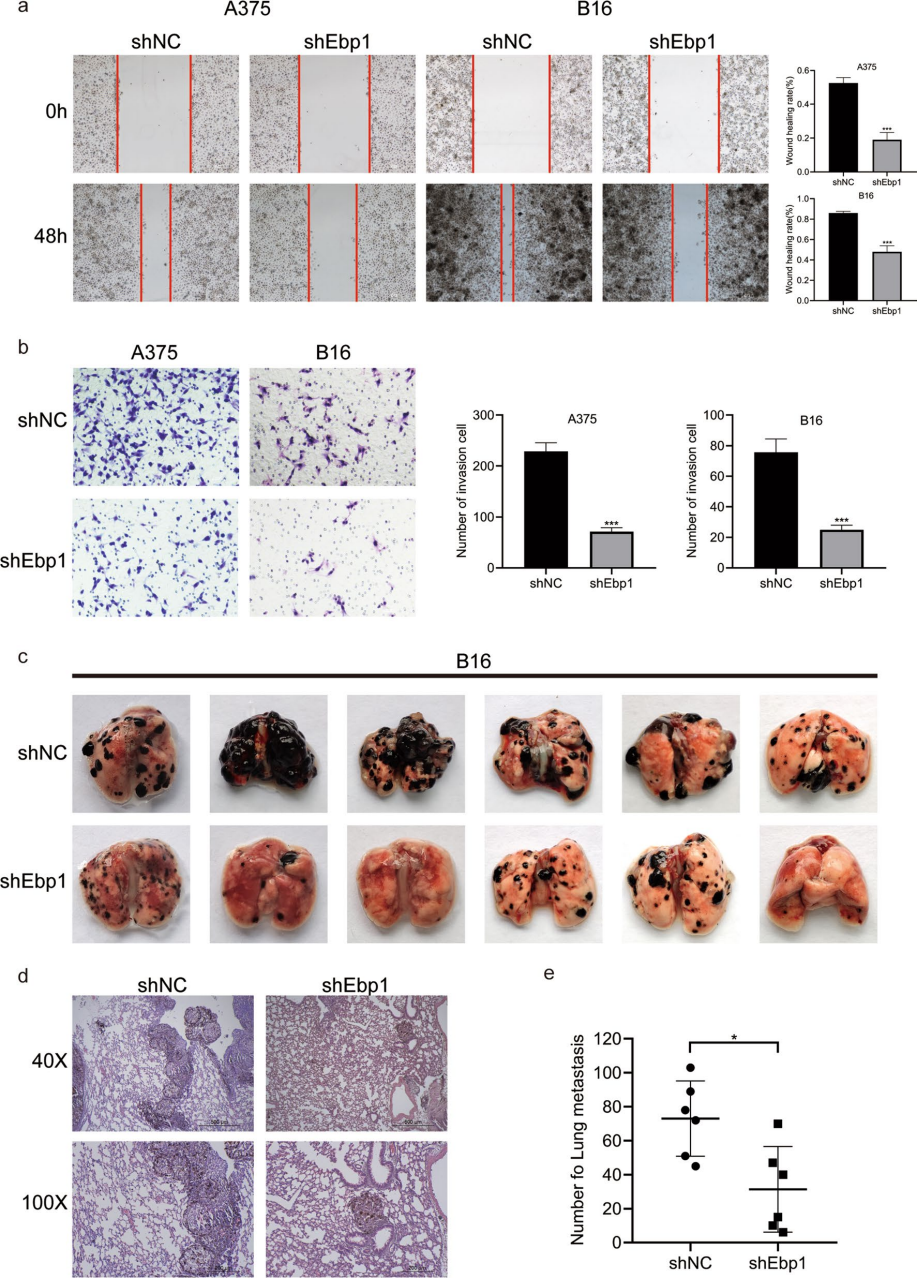
Knockdown of Ebp1 inhibits proliferation and metastasis of melanoma cells in vitro and in vivo. **a** The migration capability of A375 and B16 cells were determined via wound-healing experiments (^***^p < 0.001, vs. shNC). **b** Effect of Ebp1 on the invasion of melanoma cells by Transwell assay (^***^p < 0.001, vs. shNC). **c** Ebp1 knockdown decreases the lung metastasis ability of B16 cells. **d** Morphological feature of the lung metastasis by H&E staining. **e** Surface pulmonary metastatic nodules were counted (^*^p < 0.05, vs. shNC)

The above experiments demonstrated the effect of Ebp1 on the proliferation of melanoma cells. The effects of Ebp1 on melanoma cells invasion, migration, and metastasis were then investigated. On performing the scratch test, it was observed that the migration rate of cells in the Ebp1 knockdown group was much lower 48 h after the scratch than that of cells in the non-knockdown group (Fig. 3a). In addition, the invasive potential of cells in the Ebp1 knockdown group was much lower than that of cells in the non-Ebp1 knockdown group, according to the results of the transwell experiment (Fig. 3b). Subsequently, the nude mice were injected with B16 sh-NC or sh-Ebp1 group cells via the caudal vein. The incidence of lung metastasis in nude mice injected with sh-Ebp1 group cells was significantly lower than sh-NC group (Fig. 3c). Notably, histological examination showed that nude mice injected with the non-knockdown cells had larger and more metastatic lung nodules (Fig. 3d). These findings imply that the knockdown of Ebp1 significantly reduced the metastasis of melanoma cells.

### Knockdown of Ebp1 can inhibit EMT in melanoma cells

**Fig. 4:**
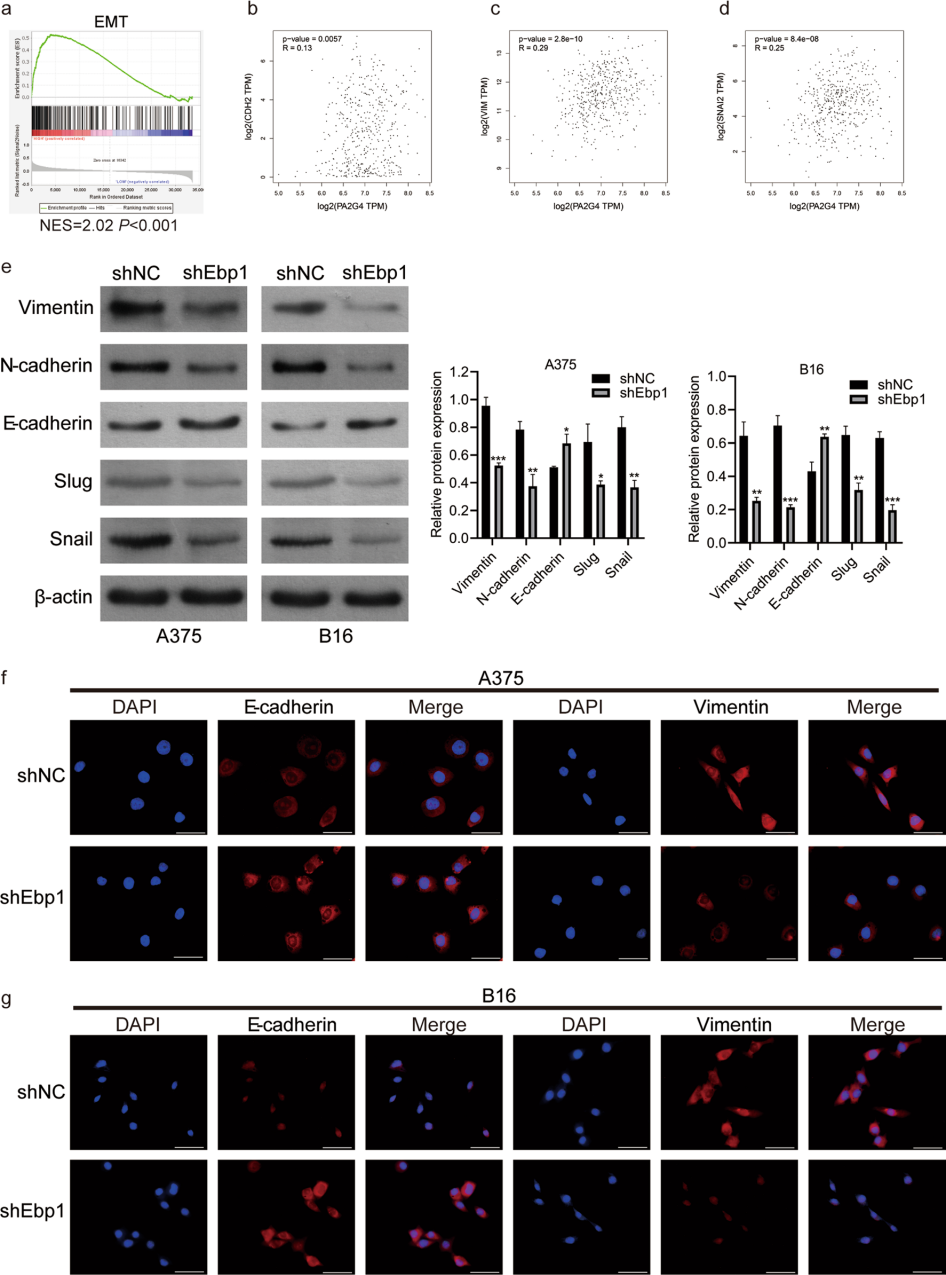
Knockdown of Ebp1 inhibited EMT in melanoma cells. **a** Ebp1 was closely related to EMT by GESA analysis. **b**–**d** GEPIA website analysis indicated that the expression level of Ebp1 was positively correlated with CDH2 (N-cadherin), VIM (Vimentin) and SNAI2 (Slug). **e** The expression of EMT related proteins was detected by western blot (^***^p < 0.001, ^**^p < 0.01, ^*^p < 0.05, vs. shNC). **f**, **g** Immunofluorescence staining assay was used to detect the expression of E-cadherin and Vimentin, Scale bar: 50 µm

Given that knocking down Ebp1 inhibits the migration, invasion, and lung metastasis of melanoma cells, and EMT is a crucial biological mechanism that gives tumor cells the ability to migrate and invade, we hypothesized that the knockdown of Ebp1 may inhibit EMT in malignant melanoma cells. First, we retrieved EMT-related genes from the gene set enrichment analysis (GESA) website and found that Ebp1 was closely related to EMT through GESA gene set enrichment analysis (Fig. 4a). Next, we used the Gene Expression Profiling Interactive Analysis (GEPIA) to analyze the correlation between Ebp1 and EMT. Ebp1 was shown to be positively correlated with CDH2 (N-cadherin), VIM (vimentin), and SNAI2 (Slug) (Fig. 4b–d). Furthermore, western blotting showed that in the Ebp1 knockdown group, the expression of vimentin, N-cadherin, Slug, and Snail protein decreased, while the expression of E-cadherin increased (Fig. 4e). Moreover, similar results were observed in an immunofluorescence experiment (Fig. 4f, g). In conclusion, Ebp1 promotes metastasis of melanoma cells by inducing EMT.

### Knockdown of Ebp1 can inhibit Wnt/ β-catenin signaling pathway

**Fig. 5:**
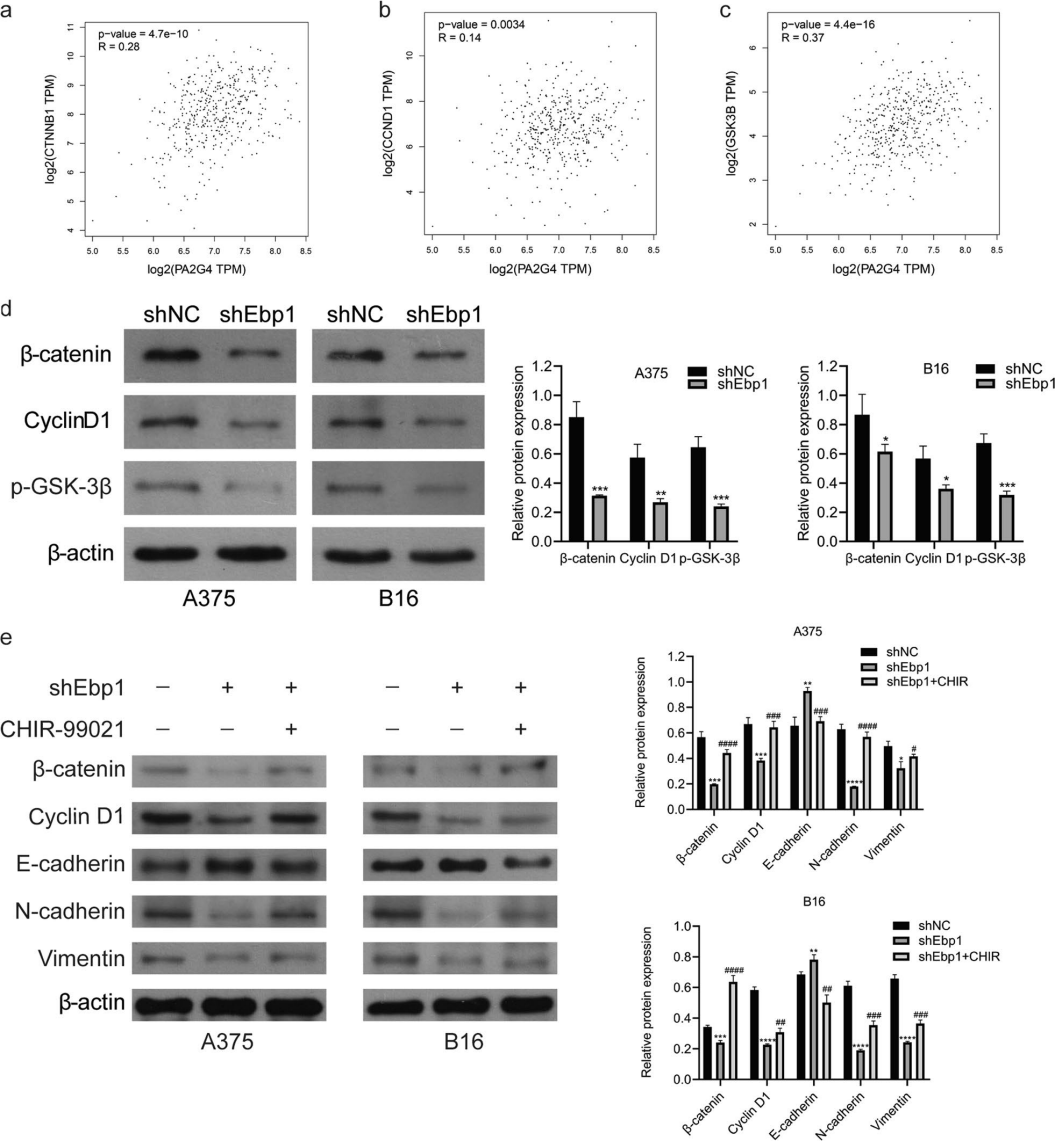
Knockdown of Ebp1 inhibited the Wnt/β-catenin signaling pathway. **a**–**c** GEPIA website analysis indicated that the expression level of Ebp1 was positively correlated with CTNNB1 (β-catenin), CCND1 (CyclinD1) and GSK3B (GSK3β). **d** Western blotting showed that knockdown of Ebp1 down-regulated the expression of β-catenin, CyclinD1 and p-GSK3β. **e** CHIR99021 reversed the changes caused by Ebp1 knockdown in related proteins. (^****^p < 0.0001, ^**^*p < 0.001, ^**^p < 0.01, ^*^p < 0.05, vs. shNC ^####^p < 0.0001, ^###^p < 0.001, ^##^p < 0.01, ^#^p < 0.05, vs. shEbp1)

One of the classical signaling pathways involved in tumorigenesis and development is the Wnt/β-catenin pathway. High β-catenin expression may induce EMT. In order to explore the mechanism of Ebp1 regulating EMT in melanoma cells, we studied the expression of β-catenin and the downstream signaling molecules in the wnt/β-catenin pathway. Through GEPIA website analysis, it was found that Ebp1 was positively correlated with GSK3B (GSK-3β), CTNNB1 (β-catenin) and CCND1 (cyclin D1) (Fig. 5a–c). Western blotting revealed that the expression of β-catenin, cyclin D1, and p-GSK-3β decreased after Ebp1 knockdown (Fig. 5d). To further explore the association between the Wnt/β-catenin signaling pathway and Ebp1 knockdown, we added 6 µM CHIR99021 to A375 and B16 cell culture medium for 24 h. Subsequently, western blotting revealed that CHIR99021 reversed the changes caused by Ebp1 knockdown in related proteins (Fig. 5e). The above results indicate that knockdown of Ebp1 inhibits melanoma cells proliferation, invasion, and metastasis through the Wnt/β-catenin pathway.

## Discussion

Although MM has a low global cancer incidence rate, the mortality rate of MM patients is significantly high owing to its high metastasis rate [19]. Early surgical resection can achieve a good curative effect; however, the treatment difficulty increases significantly in cases of metastatic MM, which causes many patients to relapse or have a poor prognosis [20]. Therefore, the mechanism of metastasis in MM requires further investigation.

The PA2G4 gene encodes Ebp1, a multifunctional protein that regulates proliferation [21]. The two subtypes of Ebp1 play different roles in different types of cancers [22]. Since Ebp1 p48 is the main form of maintenance expression in mammalian cells [23], research on the carcinogenic effect of Ebp1 p48 is critical. Ebp1 p48 promotes the malignant biological behavior of glioma cells through the negative regulation of p53, according to current research [11]. In addition, in oral tumors, Ebp1 promotes tumorigenesis by activating podoplanin [24]. Moreover, some scholars have proposed that the carcinogenic effect of Ebp1 p48 depends on the phosphorylation of the N-terminal domain of the longer subtype by CDK2 [25]. Pisapia et al. found that Ebp1 p48 was highly expressed in M14 melanoma cells, and Ebp1 p48 promoted the growth of glioblastoma cells and melanoma cells as an oncogene [26]. However, there are no reports on the mechanism by which Ebp1 p48 promotes metastasis in melanoma. Because the high metastasis rate of melanoma is the most common lethal factor, it is crucial to study the mechanism of melanoma metastasis. This study investigated the effect of Ebp1 on the progression of MM. First, by analyzing the clinical data of melanoma patients in TCGA and GTEx databases, it was found that Ebp1 was highly expressed in MM, and was linked to the disease progression and prognosis of patients with MM. This suggests that Ebp1 may be carcinogenic in the occurrence and development of MM. To test our hypothesis, we knocked down Ebp1 in two melanoma cell lines. It was found that the knockdown of Ebp1 drastically reduced the proliferation, migration, and invasion of the A375 and B16 cells. Combined with the results of the in vitro tests, we performed lung metastasis experiments in vivo. Notably, the results of the in vivo experiments show that Ebp1 plays an important role in promoting melanoma metastasis, which is consistent with the carcinogenic role of Ebp1 in glioma cells and oral tumors. In recent years, studies have shown that the ERBB receptor family, including ERBB1, ERBB2, ERBB3 and ERBB4, are important regulators of skin homeostasis. Their imbalance usually leads to cancer [27]. Moreover, the high expression level of ERBB3 significantly correlated with the poor survival rate of melanoma patients [28]. Ebp1 is an ERBB3 binding protein. We considered that Ebp1, following its interaction with ERBB3, may cause an imbalance in ERBB3 in melanoma, which leads to poor prognosis for patients with high Ebp1 expression. However, future research is needed to confirm this hypothesis.

EMT is important in carcinogenesis and metastasis [29]. Growing evidence shows that non-epithelial tumors, including MM, also have mesenchymal-like characteristics, which help the tumor cells to acquire strong metastatic ability [30]. Therefore, we speculate that Ebp1 may enhance MM cells invasion and metastasis by inducing EMT. Through western blotting and immunofluorescence, we found that Ebp1 knockdown changed the expression of EMT-related proteins. This suggests that Ebp1 may be involved in the EMT process.

The molecular mechanisms regulating EMT are diverse. EMT plays an important role in cell adhesion [31]. Studies have shown that E-cadherin usually forms a complex with β-catenin and inhibits the movement of tumor cells [32, 33]. However, E-cadherin can be down-regulated by specific transcription factors, such as the SNAIL, SLUG, and ZEB zinc finger proteins [34–36]. These transcription factors are affected by different signaling pathways, including TGF-β members, Notch, Hedgehog, and Wnt [37]. Wnt/β-catenin signaling pathway is closely related to cancer occurrence and development [38, 39]. In addition, the Wnt signaling pathway also plays an important role in melanocyte differentiation and cell cycle progression, and has also been proven to play a role in melanoma progression [8, 40, 41]. At the same time, a decrease in E-cadherin will cause intracellular β-catenin aggregation. A large amount of undegraded β-catenin transfers to the nucleus and activates downstream Wnt-dependent transcription factors, thus promoting the growth and invasion of different types of cancer cells, including melanoma [42, 43].

β-catenin is the central molecule of the Wnt signaling pathway. Under normal circumstances, the destruction complex composed of APC, Axin, GSK-3β, and CK1α induces the degradation of β-catenin in the cytoplasm [44]. Moreover, when Wnt/β-catenin pathway is activated, the destruction complex is inactivated, resulting in β-catenin accumulation in the cytoplasm and entry into the nucleus to promote downstream target genes [45–47]. GSK-3β is the main negative regulator of the Wnt/β-catenin signaling pathway [48]. GSK-3β mainly regulates the phosphorylation, degradation, and translocation of β-catenin [49]. GSK-3β decreases the amount of β-catenin in the cytoplasm, resulting in a reduction in the number of β-catenin entering the nucleus. According to the above literature, we considered that Ebp1 may affect β-catenin degradation by regulating GSK-3β. CHIR99021 is an inhibitor of GSK3β and a specific activator of the Wnt/β-catenin pathway [50]. Moreover, 6 µM CHIR99021 was added to A375 and B16 knockdown Ebp1 cells. CHIR99021 reversed the changes caused by Ebp1 knockdown in related proteins. These results suggest that Ebp1 affects the progression of malignant melanoma in a Wnt/β-catenin dependent manner.

## Conclusions

Finally, we found that Ebp1 was involved in melanoma cells' metastasis and proliferation. Ebp1 promotes malignant progression and metastasis of melanoma cells by activating EMT and Wnt/β-catenin pathways. As a result, Ebp1 may be a potential therapeutic target.

## Data Availability

The datasets used and/or analyzed during the current study are available from the corresponding author upon reasonable request.

## Abbreviations

MM: Malignant melanoma
Ebp1: ERBB3 binding protein 1
PA2G4: Proliferation associated protein 2G4
GBM: Glioblastoma multiforme
TCGA: The Cancer Genome Atlas
GTEx: Genotype Tissue Expression
GEO: Gene Expression Omnibus
EMT: Epithelial mesenchymal transformation
GESA: Gene set enrichment analysis
